# Neurotrophins and neurotrophin receptors in pulmonary sarcoidosis - granulomas as a source of expression

**DOI:** 10.1186/1465-9921-11-156

**Published:** 2010-11-08

**Authors:** Charlotta Dagnell, Johan Grunewald, Marija Kramar, Helga Haugom-Olsen, Göran P Elmberger, Anders Eklund, Caroline Olgart Höglund

**Affiliations:** 1 Department of Medicine Solna, Respiratory Medicine Unit, Karolinska Institutet/Karolinska University Hospital Solna, Stockholm, Sweden; 2 Department of Physiology and Pharmacology, Karolinska Institutet, Stockholm, Sweden; 3 Department of Oncology and Pathology, Karolinska University Hospital Solna, Stockholm, Sweden

## Abstract

**Background:**

Pulmonary sarcoidosis is an inflammatory disease, characterized by an accumulation of CD4^+ ^lymphocytes and the formation of non-caseating epithelioid cell granulomas in the lungs. The disease either resolves spontaneously or develops into a chronic disease with fibrosis. The neurotrophins nerve growth factor (NGF), brain-derived neurotrophic factor (BDNF) and neurotrophin-3 (NT-3) have been suggested to be important mediators of inflammation and mediate tissue remodelling. In support of this, we have recently reported enhanced NGF levels in the airways of patients with pulmonary sarcoidosis. However, less is known about levels of BDNF and NT-3, and moreover, knowledge in the cellular sources of neurotrophins and the distribution of the corresponding neurotrophin receptors in airway tissue in sarcoidosis is lacking.

**Methods:**

The concentrations of NGF, BDNF and NT-3 in bronchoalveolar lavage fluid (BALF) of 41 patients with newly diagnosed pulmonary sarcoidosis and 27 healthy controls were determined with ELISA. The localization of neurotrophins and neurotrophin receptors were examined by immunohistochemistry on transbronchial lung biopsies from sarcoidosis patients.

**Results:**

The sarcoidosis patients showed significantly enhanced NT-3 and NGF levels in BALF, whereas BDNF was undetectable in both patients and controls. NT-3 levels in BALF were found higher in patients with non-Löfgren sarcoidosis as compared to patients with Löfgren's syndrome, and in more advanced disease stage. Epithelioid cells and multinucleated giant cells within the sarcoid granulomas showed marked immunoreactivity for NGF, BDNF and NT-3. Also, immunoreactivity for the neurotrophin receptor TrkA, TrkB and TrkC, was found within the granulomas. In addition, alveolar macrophages showed positive immunoreactivity for NGF, BDNF and NT-3 as well as for TrkA, TrkB and TrkC.

**Conclusions:**

This study provides evidence of enhanced neurotrophin levels locally within the airways of patients with sarcoidosis. Findings suggest that sarcoid granuloma cells and alveolar macrophages are possible cellular sources of, as well as targets for, neurotrophins in the airways of these patients.

## Introduction

Sarcoidosis is an inflammatory granulomatous disease which primarily affects the lungs. The disease is characterized by an accumulation of CD4^+ ^lymphocytes and the formation of non-caseating epithelioid cell granulomas in the affected organs. The granuloma consists of highly differentiated mononuclear phagocytes (epithelioid cells and multinucleated giant cells) surrounded by lymphocytes [1]. The disease either resolves spontaneously or develops into a more chronic disease where the sarcoid granulomas develop fibrotic changes, which in the airways may lead to a progressive loss of lung function. Factors that influence granuloma formation and the development of fibrosis are not well understood in sarcoidosis [2]. Löfgren's syndrome is a form of sarcoidosis, which affects about 1/3 of Scandinavian sarcoidosis patients, and is characterized by an acute onset of disease with fever, bilateral lymphadenopathy, erythema nodosum and/or ankle arthritis [3]. Löfgren's syndrome is mostly associated with complete disease resolution, often within two years, without the need of any treatment while an insidious onset (non-Löfgren sarcoidosis) is accompanied with a higher risk of developing chronic disease with progressive fibrosis of the lungs.

We have recently reported higher levels of nerve growth factor (NGF) in the airways of patients with sarcoidosis as compared to healthy subjects [4]. NGF, brain-derived neurotrophic factor (BDNF) and neurotrophin-3 (NT-3) belong to the family of neurotrophins, and are structurally and functionally related mediators. Neurotrophins are essential survival factors for nerve cells and are critical for the development of peripheral sensory neurons [5]. However, neurotrophins and their corresponding receptors are not only expressed within the nervous system, but are also present in non-neuronal cells and in the airways [6,7]. Structural cells, like epithelial and smooth muscle cells [6-8], and immune cells, such as mast cells, eosinophils and lymphocytes [9-11], express neurotrophins as well as their receptors. NGF has been immunolocalized to fibrotic tissue in the lungs and found in elevated levels in sputum from patients with interstitial pulmonary fibrosis (IPF) [12-14]. Several studies have shown that neurotrophins have tissue healing properties, and are able to promote tissue remodelling in airway disease [8,14,15]. In addition, neurotrophins seem to have pro-inflammatory properties and mediate effects such as mast cell survival and degranulation [16], eosinophil chemotaxis [17] and lymphocyte activation [18,19]. In this context, neurotrophins have been shown to play a role in pulmonary inflammation in asthma [20]. Increased levels of NGF, BDNF and NT-3 have been found in asthmatic airways and are closely linked to airway hyper responsiveness [6,18,21,22].

While knowing that NGF is elevated in bronchoalveolar lavage fluid (BALF) of patients with pulmonary sarcoidosis, less is known about the neurotrophins BDNF and NT-3. Moreover the cellular sources of neurotrophins and the distribution of the corresponding neurotrophin receptors in the airways of these patients are poorly understood.

The aim of the present study was to compare the concentrations of the neurotrophins NGF, BDNF and NT-3 in BALF of patients with newly diagnosed pulmonary sarcoidosis with those of healthy controls. Furthermore, the aim was to identify the localization of neurotrophins, and the corresponding neurotrophin receptors, within the sarcoid lung tissue.

## Methods

### Subjects

This study included 41 patients with newly diagnosed sarcoidosis (4 current smokers, 10 ex-smokers, 27 never-smokers). All subjects had a typical clinical and radiographic picture compatible with the disease in addition to an elevated bronchoalveolar lavage (BAL) CD4/CD8 ratio and/or a biopsy showing non-caseating epithelioid cell granulomas. Diagnosis was established according to defined criteria set up by the World Association of Sarcoidosis and other Granulomatous Disorders (WASOG) [1]. Twentysix of the patients were diagnosed with Löfgren's syndrome [3]. Twentyseven never-smoking healthy volunteers with normal chest radiographs were included as healthy controls. No subject received corticosteroids at the time of BAL and blood (serum) sampling. Paired blood and BAL samples were obtained from 37 of the sarcoidosis patients and from all healthy subjects. Clinical characteristics are presented in Table 1.

**Table 1:** Clinical characteristics of study subjects included for bronchoalveolar lavage studies.

			Sarcoidosis patients	
			
	Healthy subjects	Sarcoidosis patients	Löfgren's syndrome	Non-Löfgren
**Subjects, n**	27	41	26	15
**Sex, (M/F)**	11/16	26/15	12/14	12/3
**Age, yr**	30 (24-39)	37 (31-41)	36 (29-41)	38 (32-42)
**Radiograph stage, (I/II/III/IV)**	-	22/16/3/0	16/10/0/0	6/6/3/0
**Pulmonary function tests, %**				
VC	99 (94-106)	95 (83-104)	97 (84-105)	91 (79-101)
FEV_1_	99 (92-108)*	91 (86-99)	90 (86-102)	95 (77-101)

Data are presented as medians (interquartile ranges). M, male; F, female; VC, vital capacity; FEV_1_, forced expiratory volume in 1 s. ^*^; p < 0.05 versus sarcoidosis patients

For immunohistochemistry, biopsy specimens showing non-caseating epithelioid cell granuloma formation compatible with sarcoidosis were collected from 19 additional sarcoidosis patients, diagnosed according to the above defined criteria. In 17 cases the biopsies were transbronchial, in one patient lung tissue was sampled through video-assisted thoracoscopy, and in one case an intrathoracic lymph node biopsy was obtained during mediastinoscopy. Four of the patients were current smokers, 2 ex-smokers, and 13 were never-smokers. Seven of the patients had Löfgren's syndrome.

Bronchoscopy, including BAL (see below) and biopsy sampling, were performed in all patients as they were referred to the lung clinic at Karolinska University Hospital in Stockholm, Sweden, for diagnostic purposes. The study was approved by the Regional Ethical Review Board in Stockholm (http://www.epn.se) (Dnr: 2005/1031-31) and in accordance with the Helsinki Declaration. All subjects gave their written informed consent.

### Fiberoptic bronchoscopy

BAL of sarcoidosis patients and healthy subjects was performed as previously described [23]. Briefly, a flexible fiberoptic bronchoscope (Olympus Optical Co., Japan) was wedged into a middle-lobe bronchus and five aliquots of 50 ml sterile PBS solution were instilled and re-aspirated. Recovered BAL fluid (BALF) was separated into a cell- and debris free BALF, which was stored at -70°C until analyzed, and a cell fraction from which cytospin slides for differential cell counts were prepared and analyzed as previously described [23]. Biopsies were fixed in a buffered 10% formalin solution for 24 h and embedded in paraffin.

### Analysis of neurotrophins with ELISA

Neurotrophins were quantified in BALF and serum by commercially available, two-site enzyme-linked immunosorbent assay (ELISA)-kits according to the manufacturer's instructions (Promega, USA) and as previously described [12,21]. Detection limit was 4.7 pg/ml for NT-3 and 7.8 pg/ml for NGF and BDNF ELISA kits. All samples were analyzed in duplicates and serum samples were diluted in PBS before analysis (1:100 for NT-3 analysis and 1:500 for BDNF analysis).

### Immunohistochemistry

Serial 4 μm thick sections were mounted on slides and processed for immunohistochemistry. Sections were deparaffinized in xylene, stepwise rehydrated through graded ethanol, and antigen retrieval was achieved by boiling slides in 10 mM citrate buffer (pH 6.0) (for neurotrophins and neurotrophin receptors) or ethylenediamine tetraacetic acid (EDTA) buffer (pH 9.0) (for CD68) for 20 min in microwave oven. After cooling and washing in PBS, slides were incubated in 0.3% H_2_O_2 _for 30 minutes to block endogenous peroxidase activity. After blocking with 5% goat serum or horse serum (for CD68) for 1 h at room temperature, slides were exposed to primary antibodies diluted in blocking buffer over night at 4°C. Primary antibodies and dilutions are presented in Table 2. In control experiments, primary antibodies were omitted. Non-specific binding of anti-NGF, -BDNF, -NT-3, -TrkA, TrkC and TrkB was evaluated by incubating slides with the antibodies pre-adsorbed with the corresponding blocking peptides (ratio 1:5) (Santa Cruz Biotechnology Inc, Santa Cruz, CA, USA). After incubation, slides were washed and exposed to relevant biotinylated secondary antibodies (goat anti-rabbit or horse anti-mouse) (1:300) (Vector Laboratories, Burlingame, CA, USA) for 1 h at room temp. The product of immune reaction was revealed using Vectastain^®^, Elite^®^, ABC Kit (Vector Laboratories) followed by SIGMA FAST™ 3,3 diaminobenzidine (Sigma-Aldrich, St. Louis, MO, USA). Sections were then counter-stained with Mayer's hematoxylin before they were dehydrated, mounted and viewed under light microscope (Leica DMLB) at a magnification of ×100, ×200 and/or ×400.

**Table 2:** Anti-human antibodies used for immunohistochemical staining.

Antibody	Cat No	Source	Dilution	Manufacturer
NGF	sc-548	rabbit	1:100	Santa Cruz Biotech. Inc
BDNF	sc-546	rabbit	1:100	Santa Cruz Biotech. Inc
NT-3	sc-547	rabbit	1:100	Santa Cruz Biotech. Inc
TrkA	sc-118	rabbit	1:100	Santa Cruz Biotech. Inc
TrkB	sc-12	rabbit	1:100	Santa Cruz Biotech. Inc
TrkC	sc-117	rabbit	1:100	Santa Cruz Biotech. Inc
CD68	M 0876	mouse	1:200	DakoCytomation

### Statistical analysis

Data are presented as medians (interquartile range). Mann-Whitney test, or Kruskal-Wallis test followed by Dunn's post test, were used for group comparisons. A p-value < 0.05 was considered significant. Analyses were performed with Graphpad Prism 4.03 (Graphpad Software Inc., USA).

## Results

### BAL analysis and differential cell counts

BAL recovery was higher in healthy subjects as compared to sarcoidosis patients (78; 68-79% vs. 68; 60-75%, p < 0.05). BAL cell viability was similar in both healthy subjects and sarcoidosis patients (median: 95%). BAL differential cell counts are presented in Table 3. In BALF, the total cell concentration as well as concentrations of macrophages, lymphocytes and neutrophils were significantly higher in sarcoidosis patients as compared to healthy subjects. As expected, the percentage of macrophages was lower and the percentage of lymphocytes was higher in sarcoidosis patients compared to healthy subjects.

**Table 3:** Differential cell counts in bronchoalveolar lavage.

			Sarcoidosis patients	
			
	Healthy subjects	Sarcoidosis patients	Löfgren's syndrome	Non-Löfgren
Total cell conc. *10^6^/L	81 (62-94) ***	201 (133-308)	188 (131-308)	252 (132-319)
Macrophages %	94 (91-95) ***	75 (63-84)	77 (63-86)	69 (60-80)
Macrophages *10^6^/L	75 (58-92) ***	143 (90-210)	141 (85-230)	165 (91-200)
Lymphocytes %	5.2 (3.8-7.0) ***	23 (14-34)	22 (13-33) ^#^	29 (19-40)
Lymphocytes *10^6^/L	4.4 (2.7-6.1) ***	45 (22-81)	41 (15-76)	54 (36-87)
Eosinophils %	0 (0-0.2)	0.0 (0-0.7)	0.1 (0-0.6)	0.0 (0-1.0)
Eosinophils *10^6^/L	0 (0-0.2)	0.0 (0-1.2)	0.2 (0-1.2)	0.0 (0-1.6)
Neutrophils %	1.0 (0.4-1.4)	1.0 (0.6-1.8)	1.4 (0.5-2.1) ^#^	0.6 (0.6-1.0)
Neutrophils *10^6^/L	0.7 (0.3-1.1)***	2.3 (0.9-3.5)	2.6 (1.2-4.1)	1.5 (0.9-2.6)
CD4/CD8 ratio	ND	7.3 (4.2-11)	8.2 (4.9-14)	6.9 (3.8-9.8)

Data are presented as medians (interquartile ranges). ND: not determined. ***; p < 0.001 versus sarcoidosis patients, ^**#**^; p < 0.05 versus non-Löfgren.

### Neurotrophin levels in BALF

**Figure 1:**
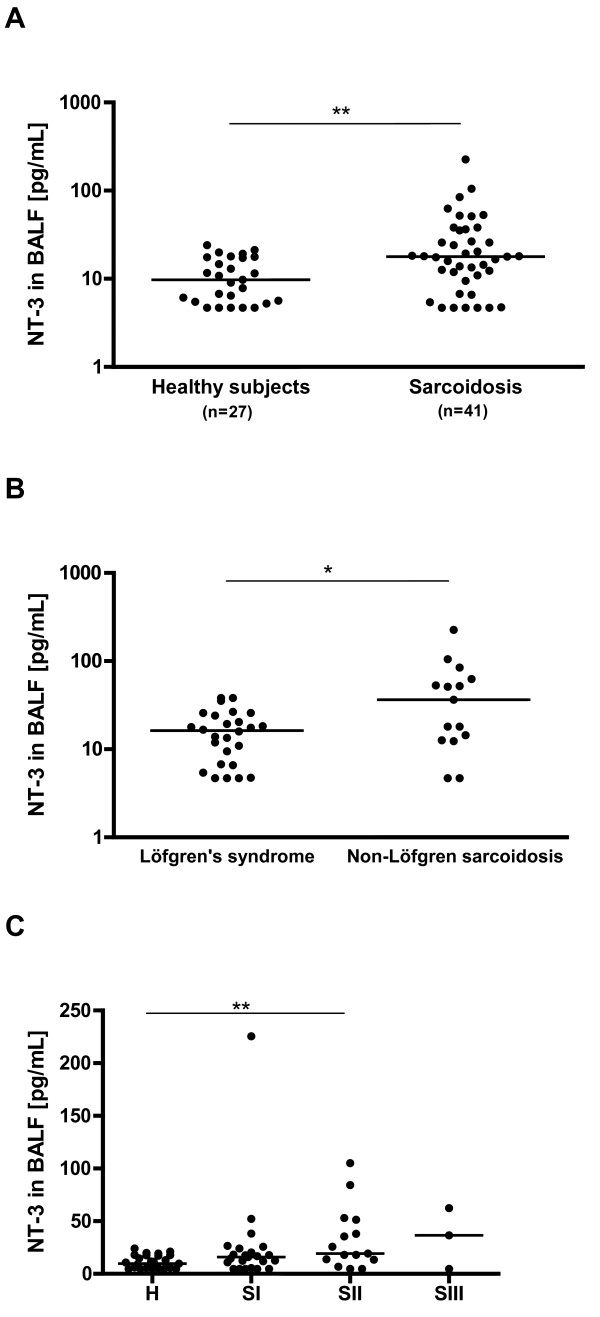
**Neurotrophin-3 (NT-3) levels are increased in sarcoidosis**. NT-3 protein levels in bronchoalveolar lavage fluid (BALF) from healthy subjects and patients with sarcoidosis (A), and in subgroups of sarcoidosis patients divided according to Löfgren's syndrome or not (B) and radiographic stage (C). Horizontal bars indicate median values. *: p < 0.05, **: p < 0.01.

Significantly elevated concentration of NT-3 was found in BALF from sarcoidosis patients as compared to healthy subjects (Figure 1A). When sub-grouping the sarcoidosis patients, significantly higher levels of NT-3 were found in BALF from patients with non-Löfgren sarcoidosis compared to patients with Löfgren's syndrome (Figure 1B). In addition, higher NT-3 levels were associated with more advanced disease stage (Figure 1C). In line with our previous report [4], NGF was significantly elevated in sarcoidosis patients (14.8; 6.1-22.6 pg/ml) as compared to healthy subjects (4.7; 2.0-17.0 pg/ml) (p < 0.01). In the present material, NGF showed no significant association with Löfgren's syndrome or disease stage. BDNF in BALF was below the detection limit of the ELISA kit.

### Neurotrophin levels in serum

No significant differences were found in NT-3 or BDNF concentrations between patients and controls or between subgroups of sarcoidosis patients. The concentration of NT-3 was approximately 3000 times higher in serum (41; 22-79 ng/ml) compared to BALF (13.0; 10.0-19.3 pg/ml). BDNF concentration in serum was 16; 12-24 ng/ml. NGF levels in serum were not determined as the levels have previously been reported by us to be below detection limit [4].

### Neurotrophin and neurotrophin receptor expression in sarcoid lung tissue

**Figure 2:**
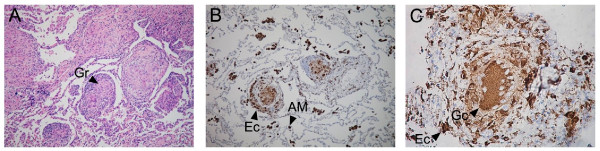
**Lung biopsy sections of sarcoidosis patients**. The presence of non-caseating epithelioid cell granulomas (Gr) with a surrounding layer of lymphocytes (hematoxylin-eosin staining, ×10) is shown in A). CD68 immunostaining in alveolar macrophages (AM), epithelioid cells (Ec) (×10) is shown in B) and CD68 immunostaining in a multinucleated Giant cell (Gc) and epithelioid cells (Ec) (×40) is shown in C).

**Figure 3:**
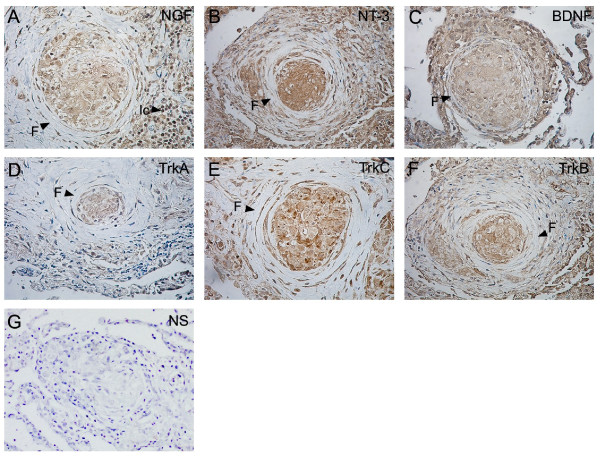
**Neurotrophin and neurotrophin receptor immunostaining in sarcoid lung granulomas**. Immunostainings for NGF (A), NT-3 (B), BDNF (C), TrkA (D), TrkC (E) and TrkB (F) on lung biopsy sections from sarcoidosis patients (×40). Positive immunostaining was localized to epitheioid cells and Giant cells within the granulomas. F: fibrosis; Ic: infiltrating inflammatory cells. Non-specific (NS) immunostaining is shown in (G) and was obtained by exposing the sections to the antibodies preabsorbed with the corresponding blocking peptide (×20).

**Figure 4:**
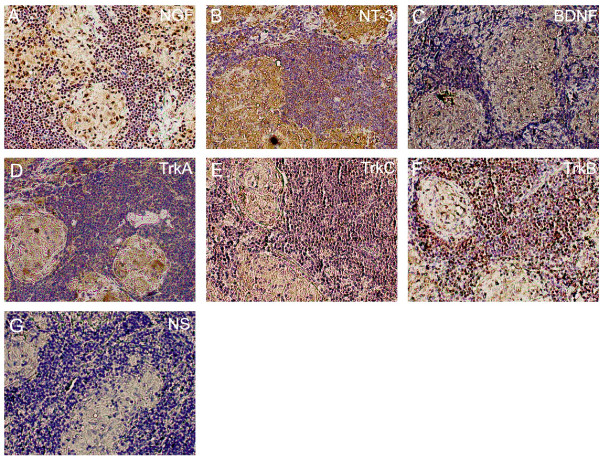
**Neurotrophin and neurotrophin receptor immunostaining in sarcoid lymph node granulomas**. Immunostainings for NGF (A), NT-3 (B), BDNF (C), TrkA (D), TrkC (E) and TrkB (F) on sections of a mediastinal lymph node from a patient with sarcoidosis (×20). Positive immunostaining was localized to the granulomas. Non-specific (NS) immunostaining is shown in G) and was obtained by exposing the sections to the antibodies preabsorbed with the corresponding blocking peptide.

**Figure 5:**
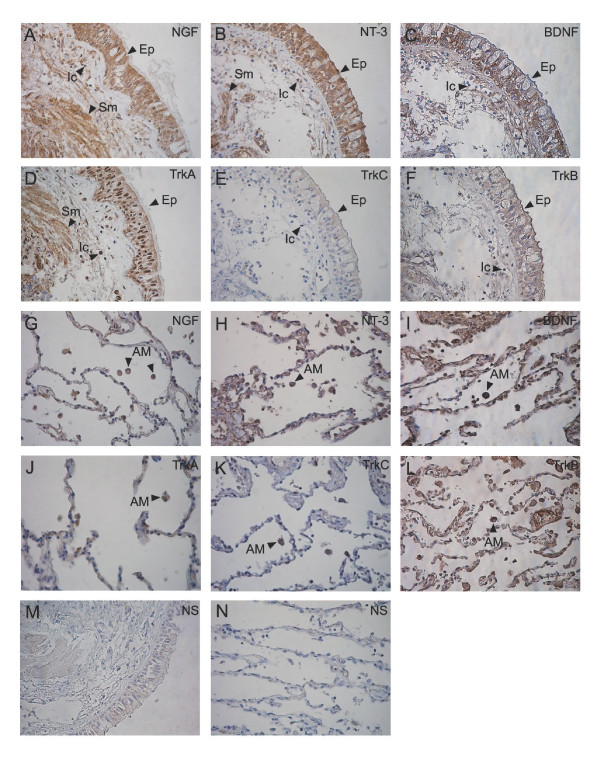
**Neurotrophin and neurotrophin receptor immunostaining in sarcoid lung tissue**. Immunostainings for NGF (A, G), NT-3 (B, H), BDNF (C, I), TrkA (D, J), TrkC (E, K) and TrkB (F, L) on lung biopsy sections from sarcoidosis patients (×20). Ep: epithelium, Sm: smooth muscle, Ic: infiltrating inflammatory cells and AM: alveolar macrophages. Non-specific (NS) immunostainings are shown in (M & N) and were obtained by exposing the sections to the antibodies preabsorbed with the corresponding blocking peptide.

Serial sections from 18 lung biopsies and one lymph node biopsy were analyzed for NGF, BDNF, NT-3, TrkA, TrkB and TrkC, respectively, by immunohistochemistry. Figures 2, 3, 4 and 5 show representative immunostainings.

The lung biopsies from sarcoidosis patients contained typical non-necrotizing granulomas composed of epithelioid cells and multinucleated giant cells and a surrounding layer of lymphocytes (Figure 2A). Strong immunoreactivity for CD68 (commonly used as a marker for monocyte and macrophage-derived cells) was found in macrophage-like cells, epithelioid cells and multinucleated giant cells (Figures 2B and 2C).

Marked NGF, BDNF and NT-3 immunoreactivity was observed in the granulomas and was localized to epithelioid cells and giant cells within the granulomas (Figures 3A, B and 3C). No or less immunoreactivity for NGF, BDNF or NT-3 was found within fibrotic tissue around granulomas (Figures 3A, B and 3C). Analysing neurotrophin receptor immunoreactivity in the tissue sections, marked TrkA, TrkB and TrkC immunoreactivity was observed within the granulomas (Figures 3D, E and 3F). Also immunoreactivity for the neurotrophins and their receptors was found in inflammatory cells surrounding the granulomas (Figure 3A and not shown).

Sarcoid granulomas in the mediastinal lymph node also showed positive immunoreactivity for NGF, BDNF, NT-3, TrkA, TrkB and TrkC, localized to the granulomas and the surrounding lymphoid tissue (Figures 4A, B, C, D, E and 4F).

Two of the lung biopsies contained ciliated bronchial epithelium and submucosa. Marked immunoreactivity for NGF, BDNF, NT-3, TrkA and TrkB, and weaker immunoreactivity for TrkC was found in the epithelium (Figures 5A, B, C, D, E and 5F). Smooth muscle cells in the submucosa showed immunoreactivity for NGF, NT-3 and TrkA (Figures 5A, B and 5D) and infiltrating inflammatory cells in the submucosa showed positive immunostaining for NGF, BDNF, NT-3, TrkA and TrkB (Figures 5A, B, C, D and 5F).

Marked immunoreactivity for NGF, BDNF, NT-3 as well as for TrkA, TrkB and TrkC was observed in alveolar macrophages (Figures 5G, H, I, J, K and 5L). The wall of the alveoli displayed positive immunoreactivity for NT-3, BDNF and TrkB (Figures 5H, I and 5L) and weaker immunoreactivity for NGF and TrkA (Figures 5G and 5J). TrkC immunoreactivity was not observed within the alveolar wall (Figure 5K).

Granulomas from patients with Löfgren's syndrome and non-Löfgren sarcoidosis showed similar localization and strength of immunoreactivity for the different neurotrophins and corresponding receptors (not shown).

## Discussion

The present study provides evidence for increased levels of the neurotrophins NT-3 and NGF locally in the airways of patients with pulmonary sarcoidosis as compared to healthy individuals. This study also describes, for the first time, neurotrophin and neurotrophin receptor expression localization in sarcoid granulomas within the lungs and lymph nodes of patients with pulmonary sarcoidosis. We have previously reported elevation of NGF in BALF of sarcoidosis patients [4], and this study confirms those results, and extends them by including the analysis of the neurotrophins NT-3 and BDNF in both BALF and serum, and by identifying cellular sources of, and targets for, NGF, BDNF and NT-3 in the lungs of sarcoidosis patients.

This study shows that not only the NGF protein, but also NT-3 protein, was elevated in BALF from sarcoidosis patients as compared to healthy subjects, while BDNF protein levels were undetectable in both healthy subjects and sarcoidosis patients. Sarcoidosis patients presenting Löfgren's syndrome have an acute disease onset and often a favorable prognosis with high rate of spontaneous disease resolution. In contrast, patients with non-Löfgren sarcoidosis run a higher risk of developing chronic disease with lung fibrosis. In this study we sub-grouped the sarcoidosis patients into those with Löfgren's syndrome and those with non-Löfgren sarcoidosis, and we could show that the levels of NT-3 were significantly higher in BALF in the non-Löfgren sarcoidosis group. Differences between subgroups of sarcoidosis patients was not seen for NGF in this study, but has been indicated in one of our previous studies, including a larger study population [4]. Chest radiographic staging (stage 0-IV) is a measurement of lung involvement in sarcoidosis and reflects disease severity, where stage 0 describes no visible intrathoracic findings and stage IV, being the most advanced stage, is characterized by pulmonary fibrosis. When sub-grouping the patients according to their radiographic stage, we found higher NT-3 levels in BALF in patients with more advances disease stage. Taken together, these results suggest an association between NT-3 and disease severity and prognosis and it could be speculated on whether NT-3 could serve as an early clinical marker for disease activity and progression in sarcoidosis.

In contrast to some other studies [24], BDNF was not possible to detect in BALF in our study. A plausible reason for this would be the difference in age of study participants, different exposure to environmental factors that may influence neurotrophin production and the fact that we used higher lavage volumes. The latter would dilute any acellular component to a larger degree. Apart from our study, also other studies have shown that the content of BDNF in BALF is near or below detection limit of the used ELISA kit [21].

In serum, concentrations of BDNF and NT-3 of healthy and sarcoidosis patients did not differ between healthy subjects and patients. Previous studies by us and others have shown that NGF concentration in serum is low or under the detection limit [4,25]. This supports the concept of a local enhancement and a possible local origin of neurotrophins in pulmonary sarcoidosis.

To elucidate the possible cellular sources of neurotrophins in the airways in sarcoidosis we performed immunohistochemistry on lung biopsy sections from distal airways of sarcoidosis patients. An intense immunostaining for NGF, BDNF and NT-3 was localized to granulomas and specifically found in epithelioid cells and giant cells of the granulomas. Epithelioid cells are macrophage-derived, highly differentiated cells with secretory functions and giant cells are multinucleated cells resulting from fusion of epithelioid cells. To our knowledge, this is the first report on neurotrophin expression in these cells and we suggest that they are possible cellular sources of the enhanced NGF and NT-3 levels detected in the bronchoalveolar lavage fluid. In our study we also had access to one pulmonary draining lymph node obtained with mediastinoscopy from a patient with pulmonary sarcoidosis. The lymphoid tissue exhibited marked granuloma formations, which were immunopositive for NGF, BDNF and NT-3. Previous reports have shown that infection-induced hepatic- and brain granulomas produce NGF, and that enhanced levels of NGF can be detected in granulomatous tissue [26-28]. Also an infectious cause has been suggested in the pathogenesis of sarcoidosis and in sarcoid granuloma formation [1].

NGF, BDNF and NT-3 immunostainings were also detected in structural- and inflammatory cells in sarcoid lung biopsies, as supported by previous studies in healthy and asthmatic airways [6,7]. Interestingly, *in vitro *studies on airway structural cells, such as epithelial cells, fibroblasts and smooth muscle cells, have shown that these cells produce neurotrophins constitutively and that the production is enhanced under inflammatory conditions [29-31]. In addition, Ricci and co-workers have demonstrated that immune cells, such as alveolar macrophages and T-lymphocytes, retrieved from BAL from sarcoidosis patients, express NGF, NT-3 and BDNF to a larger degree than BAL cells from healthy subjects [32]. We confirm and extend these results by showing positive NGF, BDNF and NT-3 immunostaining in alveolar macrophages present within the lung parenchyma of sarcoidosis patients. It is well known that T-lymphocytes and macrophages infiltrate the lungs in sarcoidosis patients, and therefore it may be postulated that an increased number of neurotrophin-expressing inflammatory cells, and specifically alveolar macrophages, in sarcoidosis patients contribute to enhanced levels of neurotrophins in BALF of these patients. Thus, we have identified multiple sources of neurotrophins in sarcoidosis airways, where the granulomas seem to be a unique source.

To elucidate the cellular targets for NGF, BDNF and NT-3 within the airways in sarcoidosis, we studied the presence of the corresponding neurotrophin receptors, TrkA, TrkB and TrkC, in lung biopsies. TrkA, TrkB and TrkC belong to the protein tyrosine kinase (Trk) family of receptors, which bind neurotrophins with high affinity. While TrkA is the primary receptor for NGF [33], TrkB is the primary receptor for BDNF [34] and TrkC is the primary receptor for NT-3 [35]. We found that the sarcoid lung granulomas were immunoreactive for both TrkA, TrkB and TrkC, indicating that neurotrophins, which are also produced within the granulomas, are able to function in an autocrine and/or paracrine manner in the granuloma microenvironment in lung tissue in sarcoidosis. Similarly, granulomas in the mediastinal lymph node were positive for the two neurotrophin receptors arguing for a possible local role of neurotrophins also in lymphoid tissue. In addition, we demonstrated TrkA, TrkB and TrkC immunoreactivity in structural cells in sarcoid biopsies, in line with previous studies in healthy airways [7,8,36]. As described previously in both healthy and sarcoid airways, we also observe neurotrophin receptor immunoreactivity in alveolar macrophages [32,37]. Taken together, the current findings suggest that, besides the granulomas, also structural- and inflammatory cells are possible targets for the neurotrophins in the airways in sarcoidosis.

Despite the lack of detectable levels of BDNF in BALF, we could detect both BDNF and its receptor in the granulomas and airway cells in sarcoidosis by immunohistochemistry in a similar fashion as for NGF and NT-3. This indicates that besides NGF and NT-3, BDNF could be a messenger molecule of relevance in pulmonary sarcoidosis. Further studies are required to support this hypothesis.

The functional roles of neurotrophins in inflammatory conditions of the airways are considered to be multiple and NGF is often referred to as an inflammatory mediator. Elevations of neurotrophins have previously been described to be linked to pulmonary inflammatory diseases, including asthma and interstitial pulmonary fibrosis (IPF). In asthma, neurotrophins have been described to be elevated in BALF as compared to healthy subjects [6,21] and to enhance airway inflammation and airway hyperreactivity [22,38-40]. Importantly, airway hyperreactivity is not a specific feature for asthma only, but is also present in patients with sarcoidosis [41]. In addition, a role for neurotrophins in wound healing and fibrosis has been suggested [42]. Thus, neurotrophin expression has been linked to airway tissue remodelling, shown to be immunolocalized to fibrotic tissue in patients with IPF [12,14], and found in increased levels in sputum of these patients [13]. Furthermore, neurotrophins have been shown to modulate fibroblast migration and pro-fibrotic phenotype [14,15]. Interestingly, we found an association of higher NT-3 levels in subgroups of sarcoidosis patients associated with a higher risk of developing chronic disease and fibrosis. Whether neurotrophin expression in sarcoid granulomas may promote the persistence of the granuloma and/or promote the development of fibrosis needs to be further investigated.

In conclusion, the present study describes that the neurotrophins NGF, BDNF and NT-3 are expressed in sarcoid granulomas in the airways and that enhanced levels of NGF and NT-3 are found in bronchoalveolar lavage fluid of patients with pulmonary sarcoidosis as compared to healthy individuals. The findings of immunoreactivity for TrkA, TrkB and TrkC, the high-affinity receptors for NGF, BDNF and NT-3, respectively, within granulomas, structural- and inflammatory cells, suggest that these are possible cellular targets for the neurotrophins in sarcoid airways. Taken together, this study supports the concept that the neurotrophins are involved airway inflammation, granuloma biology and fibrosis in inflammatory pulmonary diseases.

## Competing interests

The authors declare that they have no competing interests.

## Authors' contributions

CD performed experiments and data analysis, participated in study planning and wrote the manuscript. JG participated in the design of the study, patient recruitment, data analysis and critically reviewed the manuscript. MK performed immunohistochemical stainings and analysis. HH-O participated in study planning, patient recruitment and material collection. GE participated in study planning, patient material collection and data analysis. AE participated in study planning, patient recruitment, patient material collection and critically reviewed the manuscript. COH conceived of the study and its design, did data analysis and manuscript writing. All authors read and approved the final manuscript.
